# Using technology to enhance and encourage dance-based exercise

**DOI:** 10.1016/j.heliyon.2019.e01241

**Published:** 2019-03-07

**Authors:** Alethea Blackler, Shital Desai, Levi Swann, Marianella Chamorro-Koc, Gene Moyle, Mikaela Stephens

**Affiliations:** a Queensland University of Technology, Australia; b AGE-WELL, Inclusive Media and Design Centre - Ryerson University - Ted Rogers School of Management, Canada^1^; c Bolton Clarke, Australia

**Keywords:** Public health, Computer science

## Abstract

This study investigated the role of Self-Service Technologies (SSTs) in dance-based exercise in order to begin exploring the motivations behind the use (or not) of SSTs by ordinary men and women in this context. The research approach employed interviews to gain insights into participants' use of SSTs and their exercise practices, in order to start establishing ways in which dance can be re/incorporated into people's lives through the design of appropriate SSTs. Findings from this study highlight the significant opportunity to further explore how the properties of music and dance can be integrated into the design of new SSTs. Literature suggests dance could be a beneficial exercise format for many people and self-service technology abounds for exercise but is often not used consistently. Our interviews asked participants about dance-based exercise and SSTs for exercise and showed that there is an opportunity to design SSTs to help people access dance-based exercise. SSTs should help people learn dance, build confidence, and dance alone or with others. SSTs could facilitate movement and increase engagement with physical activity whilst addressing issues around logistics, confidence and dance knowledge and experience.

## 1 Introduction

Music and dance are an important part of the human experience. Many people engage in these activities in a social and recreational capacity. Less known and less understood, however, is that dance can also be an effective form of exercise (Angioi et al., 2009; Domene et al., 2014; Flores, 1995). Regular exercise can improve physical and cognitive function and psychological well-being, and dance-related exercise can provide similarly positive effects on physical and mental health (Angioi et al., 2009).

Despite access to extensive knowledge and information to promote health and wellbeing, health problems are pervasive in society. A contributing factor to these health problems is the increasing prevalence of inactivity in the workplace and in recreational activities (Martin et al., 2015; Ng and Popkin, 2012; Vandewater et al., 2004). In efforts to address these problems and re-engage people with physical activity, an increasing number of self-service technologies (SSTs) have been developed. SSTs are services that the customer generates and interacts with in replacement of a traditional face-to-face service (Curran and Meuter, 2005). Popular SSTs for fitness include apps for mobile devices and wearable devices such as Fitbit, Garmin, Apple Watch, and Polar heart rate monitors. These apps and devices aim to encourage healthy behaviour by prompting physical activity and monitoring progress toward wellness goals.

Although the potential benefit of SSTs for fitness is large, they often fail to achieve their aims. The effectiveness of fitness trackers has been found to be dependent on the goals set by users and their motivation to engage with the device and associated website or application (Wang, 2014; Delgado, 2014). However, apps are a crowded market, many are not downloaded, and others are downloaded and abandoned after only a short period; according to a global study of more than 300 mobile apps and 300 million user profiles, only 25% of apps in all categories are used again after the first day and fewer than 10% continue use beyond seven days. Mobile gaming apps fare slightly better with nearly 40% used beyond the first day (Greenan, 2016). Given these circumstances, the purpose of this research was to investigate how dance as an engaging type of exercise can encourage the effective use of SSTs to help people achieve their fitness goals.

## 2 Background

This section contains a thorough exploration of the issues that can be brought together to create a greater understanding of how emerging self-service technologies could assist in increasing levels of physical activity, specifically in terms of dance. This section firstly explores engagement as a concept and as applied to technology, then explains the benefits of dance and finally details the small amount of existing research on SSTs applied to dance-related exercise.

### 2.1 Engagement

The notion of engagement has been explored in several activities and domains. These include education and learning environments (Douglas and Hargadon, 2000; Strean, 2011; Williams and Germain, 2008), interactions with technology and the Internet (Lehmann et al., 2012), fitness and sport (Gao, 2012; Stålnacke Larsson, 2013), social interaction and team building (Mueller et al., 2003) and videogames (Boyle et al., 2012; Márquez Segura et al., 2013). Because of the diversity of these different activities and domains, it can be difficult to establish a clear definition of engagement. O'Brien and Toms (2008) defined engagement as:

*...a quality of user experiences with technology that is characterized by challenge, aesthetic and sensory appeal, feedback, novelty, interactivity, perceived control and time, awareness, motivation, interest, and affect.* (p.949).

While it is certainly possible to identify a number of properties of engagement that are consistent in most domains, the effects of these properties have also been shown to vary among contexts (O'Brien and Toms, 2008). To facilitate a detailed understanding of engagement, this section will discuss some of the key properties of engagement, as well as explore how these properties may differ in relation to context.

The properties of engagement can be classified as either psychological factors or technological factors (Turner, 2014). Psychological factors are concerned with the internal forces that affect engagement. These forces are generally unique to an individual and include concepts such as presence, intrinsic motivation, feelings, knowledge, interests, attention, and willingness to follow rules and participate. In contrast, technological factors describe external forces that affect engagement. These factors are typically concerned with those from other people, the environment, and relevant activities. However, it should be noted that psychological factors that are internal to a person also have some moderating influence. For example, motivation and personal interest can impact on how external forces are perceived (O'Brien and Toms, 2008).

O'Brien and Toms' (2008) model of engagement shows a process containing five potential stages of engagement with online technologies: the point of engagement, period of engagement, disengagement, re-engagement and non-engagement. These stages are important for contextualising the concepts associated with engagement and form an effective way to discuss the technological properties of engagement.

The point of engagement is associated with motivation and goals (O'Brien and Toms, 2008; Turner, 2014), where engagement is facilitated by experiences that are seen to be meaningful and relevant (Williams and Germain, 2008). For instance, a person may engage in an activity because it provides some type of progression towards a higher goal, such as participating in a training program required for a promotion at work, or to avoid an undesirable outcome, such as punishment (Ryan and Deci, 2000). Alternatively, a person may engage due to intrinsic motivation - defined as the willingness to engage for one's own sake. Intrinsic motivation describes the outcome of several psychological properties. Some of these are enjoyment (Gao, 2012; Ryan and Deci, 2000), fun (Strean, 2011), personal improvement and mastery (O'Brien and Toms, 2008), autonomy and control (Domene et al., 2014).

Other common external forces of engagement at the point of engagement can be broadly defined as concepts intended to attract and capture attention (O'Brien and Toms, 2008; Turner, 2014). In learning environments, such attractors have been identified as fun, novelty and humour (Strean, 2011; Williams and Germain, 2008). Video gamers commented on specific stimuli such as trailers or advertising as types of attractors. These elements provide exposure to information such as gameplay, story and graphics, which all contributed to excitement and anticipation about the game (O'Brien and Toms, 2008). Similar concepts are described in web and other digital products, with aesthetics and graphics having a substantial impact on engagement (Boyle et al., 2012).

Following the point of engagement is ‘the period of engagement’ (O'Brien and Toms, 2008). This stage describes the period of continued or sustained engagement during an activity and is commonly associated with the concept of immersion. The exact properties that facilitate immersive and sustained experiences of engagement differ among contexts. In the task of reading, immersion is associated with familiarity and the possession of relevant schemas to facilitate effective interpretation of the text (Douglas and Hargadon, 2000). Similarly, for online web applications, engagement is facilitated by experiences that are easily understood and do not result in confusion or frustration (O'Brien and Toms, 2008). Unlike these two examples, immersion in video games is associated with experiences that are positively challenging and test the user's skill. However, it is also important that users believe they have the requisite skill, or can obtain the requisite skill, to meet the challenges of a particular situation (Bianchi-Berthouze, 2013; O'Brien and Toms, 2008). These types of positive feelings - previous successes, adequacy and accomplishment are also important in exercise and fitness domains, and social situations (Yee, 2006; Gao, 2012; Ryan and Deci, 2000).

Disengagement occurs when a person decides to stop an activity or when external factors force them to stop. Disengagement may occur if a website is too difficult to use (O'Brien and Toms, 2008), a text is too complicated or unfamiliar (Douglas and Hargadon, 2000), or a videogame too difficult or not difficult enough (Bianchi-Berthouze, 2013; O'Brien and Toms, 2008). In relation to internal factors that may cause disengagement, an important property is feedback. Ryan and Deci (2000) found that receiving positive feedback enhanced intrinsic motivation, while negative feedback resulted in diminished intrinsic motivation. These effects have also been found to be mediated by perceived confidence, where negative feedback had less of an impact on people with higher perceived confidence (Vallerand and Reid, 1984 in Ryan and Deci, 2000).

Just as it is possible to become disengaged, it is also possible to become re-engaged. The factors contributing to re-engagement are similar to those at the point of engagement. However, an important additional facilitator of re-engagement is positive past experience. In the context of video games, O'Brien and Toms (2008) associate positive past experience with situations such as replaying a videogame because it had an enjoyable story, or simply because it was associated with a positive memory. Positive past experience can also be considered in terms of previous successes and failures or mastery experiences (Gao, 2012). Ryan and Deci (2000) state that people generally want to engage in activities that make them feel adequate, capable, effective, and in control. Gao (2012) found that when playing fitness-focused videogames, those with successful mastery experiences and positive past experience demonstrated significantly higher intrinsic and extrinsic motivation. In comparison, if a person has had a bad experience or has failed in the past, s/he may believe s/he lacks the relevant skills and will be reluctant to re-engage with an activity or situation.

The final stage of engagement identified by O'Brien and Toms (2008) is ‘non-engagement’. As hard as designers or providers may try and as encouraging as the context might be, some people may not be engaged at all. A key factor of non-engagement is amotivation, which refers to a lack of intention and absence of motivation (Gao, 2012; Ryan and Deci, 2000). Amotivation can be linked to some of the properties described above, such as repeated negative feedback or negative past experience. If an activity is perceived to have no benefit a person may choose not to participate in it (Williams and Germain, 2008), or amotivation might simply arise from having preferable options. O'Brien and Toms (2008) describe this situation in reference to online experiences, where a person may choose not to engage in online shopping because they have the alternative to shop at a physical store.

This section has provided some consideration for the focus of this research, which is to explore how we can increase engagement with SSTs to increase individual's levels of physical activity. Dance has enormous potential for facilitating movement as it embodies many of the properties that facilitate effective engagement – including minimal skill requirements coupled with opportunities for mastery of skill (Flores, 1995), self-regulation of activity and autonomy (Ryan et al., 2009; Domene et al., 2016), social interaction (Keyani et al., 2005), and enjoyment or fun (Domene et al., 2014; Flores, 1995). For these reasons, dance has received some interest in the research community for its potential to engage individuals in physical activity (Domene et al., 2014, 2016).

### 2.2 Benefits of dance

Much of the literature investigating dance and its effects on cognitive function and physical health has focused on dance as a form of ‘therapy’ for the elderly or unwell, or on the existing professional dance community. A distinct lack of research has investigated dance in the ‘general community’ (Campion and Levita, 2014; Cota et al., 2015; Kloos et al., 2013).

Of the research that has investigated dance as a viable form of exercise, results generally show that it provides many of the same benefits as traditional exercise. In a study investigating salsa dance for exercise, it was found that more than half the time spent dancing could be classified as moderate-to-vigorous physical activity (Domene et al., 2016). Similarly, an earlier study by the same researchers showed that over a two-hour period of dancing, two thirds was classified as ‘active time’ and yielded just under 10,000 steps for both men and women. 10,000 steps per day is considered a reasonable total for adults to successfully reach the recommended amount of physical activity (Domene et al., 2014). Another study (Flores, 1995) investigating the potential of dance as a type of exercise for high school students showed similarly positive results. Results showed significant decreases in BMI and heart rate in girls who participated in a dance program when compared to girls in the control group, who participated in standard physical education classes (Flores, 1995).

Beyond physical health benefits, participation in dance activities is also beneficial to mental health and well-being. Domene et al. (2016) recorded physiological experiences during Zumba and Salsa dance activities using a wrist-worn accelerometer and captured psychological experiences using a Likert-style questionnaire. Zumba fitness rated higher than Salsa dance in terms of energy expenditure and step count. Participants also reported substantial improvements in positive-well-being and psychological distress when performing Zumba. However, participants reported the highest reduction in psychological stress and depression when performed partnered Salsa dancing (Domene et al., 2016). In another study, significant enhancements to mood and self-assessment of physical appearance and emotional experience was achieved after participating in a 60-minute aerobic dance activity (McInman and Berger, 1993). Engagement in dance has also resulted in improved attitudes toward physical activity, as demonstrated by the girls who participated in Flores's (1995) dance program described earlier. Similarly, in a study by Wagener et al. (2012), improved attitudes toward physical activity that developed after of participating in dance-based exergaming were shown to increase the likelihood to continue exercising.

As a non-traditional type of exercise, dance is an attractive option for groups of people who face challenges when it comes to participating in exercise. For example, older people can require that exercise programs and technologies are designed to their own usability and engagement criteria so that they will be appropriately motivated to participate. Cruz-Ferreira et al. (2015) evaluated the effects that a regular integration of creative dance had on the physical fitness and ‘life satisfaction’ on 57 women aged 65–80, over the course of 25 weeks. Results showed that they had increased physical fitness and life satisfaction compared to the control group, and suggested that dance-based exercise can be accepted by an older demographic (Cruz-Ferreira et al., 2015). Dance-based exercise programs have also been successful in engaging young children with exercise. For example, when children were engaged in dance activity twice a week, although teachers reported that it was sometimes a struggle to get the children moving, children were found to be more focused, less agitated, and appeared to be more calm and ready to learn (Parnell, 2015). The next section explores how music might contribute to the benefits of dance described in this section.

### 2.3 Music

The outcomes of positive experience, enhanced mood, and improved feelings of well-being discussed in the previous section demonstrate the potential of dance for facilitating engagement in physical activity. Much of this potential relates to the properties of dance connecting with the sensual, emotional and spatiotemporal experiences that are instrumental for engagement. For instance, sensual experience is associated with visual, auditory and interactive components of an activity; emotional experience is associated with affective experiences such as fun and enjoyment, and also relates to physiological responses such as heart rate and ‘goose bumps’; and, spatiotemporal experience pertains to the time and space of an experience, such as perception of time, internal states and external environments (O'Brien and Toms, 2008). In the context of dance, these aspects of human experience are explored in the theory of music, mood and movement. This theory is based on the premise that music, mood and movement are closely intertwined, with each element having a profound effect on the other, thus affecting the different aspects of human experience (Murrock and Higgins, 2009).

Music is the central element of the music, mood and movement theory due to its ability to alter mood states and facilitate movement. Murrock and Higgins (2009) state that music produces psychological and physiological responses. This connection between music and movement is attributed to the spatiotemporal characteristics that are shared by music and physical motion. Similarities in terms of speed, rhythm, and smoothness engage the same brain circuits, particularly those involved in time-keeping, sequence learning and motion perception (Fan, 2013). This connection also extends to mood. When humans physically move to a beat (even simply bobbing the head), the auditory and motor areas of the brain synchronise to produce brain waves which parallel a powerful emotion of enjoyment (Fan, 2013).

Murrock and Higgins (2009, p. 6) state that “the psychological response of altered mood and the physiological response of movement to music promotes the initiation and maintenance of physical activity leading to improved health.” Not only does music facilitate movement, it maintains movement and has a substantial effect on prolonging the stage of engagement in physical activity. Incorporating music into fitness programs is believed to improve athletes' adherence to their program (Karageorghis et al., 2012); music has the capacity to increase time to exhaustion by around 18% when used during physical activity (Terry et al., 2012), and it can also reduce perceived effort, which gives credence to the emerging popularity of exercise-to-music classes such as Zumba.

### 2.4 Integrating dance and self service technologies

The benefits of dance, and its relationship to physiological and psychological wellbeing, have been clearly illustrated. Dance has been shown to elicit physiological response, emotional arousal, and positive affect. In the context of health and well-being, music and dance offer potential for enhancing engagement. These combined factors present a significant opportunity to explore how the properties of music and movement can be integrated into the design of SSTs with the purpose of increasing engagement with physical activity.

Integration of dance, music and technology is not an entirely new idea. Many apps and other digital media have been designed to enhance engagement and encourage physical activity. A common strategy used for digital media to enhance engagement with physical activity is the use of ‘gamification’. Gamification is defined as the addition of game-like elements, such as incentives or rewards for specific behaviours or achievements (Lister et al., 2014). An exercise activity with the addition of gamification elements is known as an ‘exergame’. In the case of mobile phone apps, the addition of game elements to enhance engagement in exercise is often based on including a reward and achievement system such as receiving points for reaching a fitness goal (Bogost, 2005). Other apps use novel methods such as adding story elements to enhance physical activity. An example of this is the app Zombie Run (Six to Start and Alderman, 2015), which provides a narrative in which the runner is a character escaping from zombies. The game works in any location and provides various cues that enhance physical activity, as runners have to speed up to escape zombies.

Beyond achievements and rewards, exergames can also be highly interactive, with the exercise activity directly integrated into the game. This strategy is used in many games available on the Sony Playstation, Microsoft Xbox and Nintendo Wii. Exergames for these consoles (also referred to a “controller apps”) require a specialised input device that is able to measure body movement and apply it in the game. Games that use these technologies are becoming increasingly popular, with effective integrations of workout programs ranging from moderate to intensive (Bronner et al., 2016). One of the most well-known exergames is Dance Dance Revolution (DDR). DDR requires participants to follow onscreen dance instructions, which are input into an interactive pad by performing real dance and foot movements. These interactions involve full body movement and are enhanced by the use of energetic music and visuals (Gao, 2012).

Since DDR, there has been a host of similar games released including Just Dance (Ubisoft, 2015), Singstar Dance (SCEA, 2015), and Dance Central (Harmonix, 2015). It is believed that interactive dance games provide an engaging environment to facilitate similar cognitive and emotional benefits, as well as physical benefits that are associated with body movement (Bianchi-Berthouze, 2013). However, some studies (e.g. Gao, 2012; Gao et al., 2013) have suggested that although interactive dance games effectively enhance motivation for physical exercise, they are not quite able to replace traditional types of physical exercise. Likely their greatest strength is in motivating those with sedentary ‘gaming’ lifestyles (Bronner et al., 2016).

Exergames, predominantly DDR, have been the focus of several studies investigating their viability as platforms for exercise and promoting well-being. Kloos et al. (2013) investigated the potential of dance exergames in therapy for people living with Huntington's disease. Participants used the DDR game two days per week for six weeks, and showed significant reductions in need for physical support for walking. Participants also stated that they enjoyed playing the game and wanted to continue playing after the study completion. Another study using DDR has explored the effect dance-based exercise can have upon severely overweight children (Mealey et al., 2010). Their study measured the energy expenditure and enjoyment recorded by 20 children using various exercise aids. Results showed no significant difference in energy expenditure between the different methods of activity, however, the enjoyment ratings differed significantly. DDR was rated at the highest level of enjoyment, followed by an in-home walking video, with a treadmill placing last.

It has been shown through several studies of dance-based exergames that players rate enjoyment during exergames more highly compared to traditional forms of exercise such as cycling or walking. The technology must be engaging and challenging for the participant, so that they feel motivated to return and discouraged from cutting corners with the input devices (Bronner et al., 2016). The physical exertion during even 30 minutes of dance-based exergames is well above the average for ‘standard physical activity’ and can be a viable option to keep people moving and healthy. Dance exergames promote a fun and competitive platform for participants, however, they battle the image that they are purely game based, and not consistently used as a regular form of exercise.

### 2.5 Summary of literature

Currently, the literature appears to have multiple gaps in the area of SSTs for dance-based exercise, including integration into studies of both males and females of various ages, and partnered and non-partnered dance exercise. Research has only just begun to explore this phenomenon, with many studies only analysing short-term effects on fitness and mood. There is little to no literature on the effects dance-related exercise can have long term. Most of the studies assessing the effectiveness of exergames on wellness and health focus only on energy expenditure in children and young adults (Bronner et al., 2016), with some focus on older people with particular health conditions (e.g. Kloos et al., 2013). There is also a distinct lack of research on repeat engagement with these dance-based exergames, with a trend instead focussing upon one-off sessions and/or set time periods. Finally, only exergames have been investigated in this space, with other types of SSTs (e.g. personal devices such as fitbits, watches and phones) not so far supporting dance-specific exercise programs beyond step counting. Despite these shortfalls, the extant literature has consistently shown that dance-based fitness facilitates energy expenditure comparable to traditional exercise methods, as well as a high level of enjoyment by participants.

## 3 Design

Thus, instead of implementing another intervention, this research study aimed to explore people's attitudes to engagement or potential engagement with dance-based exercise facilitated through SSTs by focussing on the following two research questions:

*RQ1 What is the role of dance in people's wellbeing?*
*RQ2 What are the attributes of engagement, disengagement, sustained engagement, and reengagement with SSTs in the context of dance-based exercise?*

## 4 Method

Eleven participants were recruited through a university email list, and through Facebook. The research was approved by the QUT University Human Research Ethics Committee (UHREC) and people who expressed their interest in participating were provided with the approved Participant Information and Consent Forms. No incentives were offered to the participants.

We made an effort to recruit “ordinary men and women” (Bakardjieva and Smith, 2001) rather than self-tracking enthusiasts in the Quantified Self community (so-called QSers), who seek self-knowledge through self-tracking variables such as heart rate, respiration, steps taken, or hours slept, in order to find personal meaning in personal data (QS Institute, 2016). Ordinary people are likely a much more realistic representation of what most people are really doing with their SSTs (Didžiokaitė et al., 2017). A participant overview is provided in Table 1.

**Table 1:** Participant demographics.

Participant	Age	Gender	Occupation	Dance as a form of exercise
P1	26	Male	Structural Engineer	School dance lessons
P2	44	Female	Counsellor	Aerobics, Dance DVDs, Just Dance and Zumba exergames
P3	23	Female	Paramedic	Zumba, contemporary
P4	66	Female	Administration	Just Dance exergame, Tango, Flamenco
P5	24	Male	Electrical Linesman	None
P6	23	Male	Student	None
P7	26	Male	Salesperson	Paid dancer
P8	52	Female	Administrative assistant	School dance lessons
P9	56	Male	Training Manager	Zumba
P10	31	Female	Homemaker	Step and Pump class
P11	34	Female	Electronic Research Assistant	Hip Hop, disco, jazz, contemporary, musical theatre

A semi-structured interview technique was selected, using broad, open-ended questions designed to draw out participants' experiences and values in the context of dance (Appendix A). The same questions were used for each participant, however, the order that questions were asked was changed depending on the answers given by the participant. This was done at the discretion of the interviewer to maintain a constant flow of discussion, and to enable a comfortable interview environment for the participant. Interviews were conducted at a preferred location identified by each participant. Locations ranged from public libraries to university conference rooms. The interviews were conducted one-on-one, and were audio recorded.

## 5 Analysis

The audio recordings of interviews were transcribed and coded using Atlas.ti. This software is ideal for text-based coding as it allows flexible use of almost any coding scheme but also facilitates basic quantification of data – e.g. the co-occurrences and frequencies that we calculated in this study. In this case the ability to find co-occurrences in the data was the most important function of the software.

Firstly, four key interview questions were coded and quantified for positive or negative responses, which are given below. The numbers further help us explain participants' responses to other questions, as discussed in the results.

1. Do you believe technology is effective in assisting your exercise?
2. Would you consider dancing as exercise?
3. Are you more inclined to exercise with music playing?
4. Would you prefer to dance alone or with a partner?

Then, O'Brien and Toms' (2008) model of users' engagement with technology was used to develop the coding scheme (Table 2). Engagement, disengagement, and sustained engagement with SSTs, and dance formed the themes for this study. The attributes for each of these themes were derived from the interview transcripts. Initial analysis of the data did not reveal information on participants' re-engagement with SSTs, exercise and/or dance. Thus, re-engagement was not included as a theme in the coding.

**Table 2:** Themes identified for coding interview transcripts (based on O'Brien and Toms' engagement framework).

Themes	Description
Point of Engagement with SSTs	Initiation and introduction of use of SSTs
Disengagement with SSTs	Complete discontinuation of the use of SSTs
Sustained engagement with SSTs	Continuous use of SSTs without disruptions or breaks
Point of Engagement with Dance	Initiation and introduction of some of form of dance
Disengagement with Dance	Complete discontinuation of dance
Sustained engagement with Dance	Continuous involvement with some form of dancing without disruptions or breaks
Point of Engagement with Exercise	Initiation and introduction of some of form of exercise
Disengagement with Exercise	Complete discontinuation of exercise
Sustained engagement with Exercise	Continuous involvement with some form of exercise without disruptions or breaks

Finally, the coded data were queried for the two research questions using the co-occurrence query tool in Atlas.ti. To find an answer to RQ1, the co-occurrence tool in Atlas.ti was run on the themes Point of Engagement with Dance and Engagement with Exercise, resulting in the sub-theme Point of Engagement with Dance as a form of Exercise. Similarly, sub-themes were created for Sustained Engagement with Dance as a form of Exercise, and Disengagement from Dance as a form of Exercise. RQ2 was answered by running the co-occurrence tool in Atlas.ti on the sub-theme Point of Engagement with dance as a form of Exercise and the theme Engagement with SSTs, resulting in a sub-theme Point of Engagement with SSTs for Dance-based Exercise. Similarly, sub-themes were created for Sustained Engagement with SSTs for Dance-based Exercise, and Disengagement with SSTs for Dance-based Exercise.

## 6 Results

**Fig. 1:**
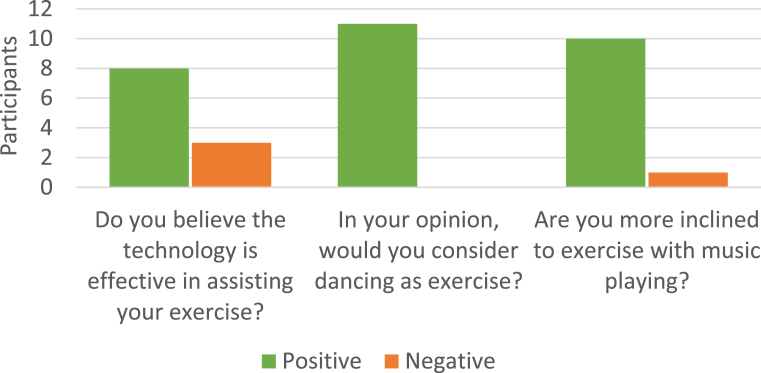
Positive and Negative responses to three key interview questions.

The occupation and age of each of the participants is shown in Table 1, along with the dance forms that they had engaged in at some point in time, and it can be seen that the participants had no expertise in technology-related design and development and no professional dance training. Thus, participants did not necessarily appreciate all the possible roles that technology could play in exercise, and especially in dance-based exercise. As such, participant responses pertaining to the use of SSTs were generally limited to their previous experience of counting steps, heart rate and other measures, tracking progress and providing feedback. SSTs used by the participants in exercise (with and without dance) were apps, DVDs, Fitbits, heart rate monitors, and controller apps (the term controller apps refers to games and apps for gaming consoles such as Xbox and PlayStation). Generally, the researcher had to explain the concept of SSTs to the participants at the point in the interview at which they were first mentioned. Fig. 1 shows positive or negative responses to key questions 1–3. All participants believed dance is exercise, eight believed SSTs were effective in assisting them to exercise, and ten would be more inclined to exercise with music playing (the other participant preferred to watch news on the TV while exercising). In addition, when asked if they would prefer to dance alone or with a partner (key question 4) only one said she would prefer to dance alone and that was due to her feeling that her husband was a bad dancer. One would prefer a group context where the whole group is working towards something, and one replied a bit of both. The other eight would all prefer to dance with a partner, as they did not want to be alone on the dance floor, felt dancing alone was weird or dancing with a partner was more fun or sociable. However, one of the eight mentioned that to dance with a partner, the partner had to be a capable dancer.

### 6.1 Engaging with dance-based exercise

The attributes that were revealed from the analysis as contributing to engagement, sustained engagement, and dis-engagement from dance-based exercise are summarised in Table 3 and discussed below.

**Table 3:** Attributes contributing to engagement, sustained engagement and disengagement with dance as a form of exercise.

Attributes for point of engagement with dance-based exercise	Intrinsic Motivation
	Extrinsic Motivation
	Enjoying the music or particular music style
Attributes for sustained engagement with dance-based exercise	Fun
	Skill, experience, and confidence in dancing
	Social setting
Attributes for dis-engagement from dance-based exercise	Lack of skill, experience, and confidence in dancing
	Problems with social setting
	Logistics of attending dance classes

The co-occurrence tool when applied to the two themes Engagement with Dance and Engagement with Exercise resulted in three attributes for engagement with dance as a form of exercise: intrinsic motivation, extrinsic motivation and enjoying the music or music style. Participants introduced some form of dance (shown in Table 2) in their exercise regime because of intrinsic motivation to remain physically and mentally fit, and for personal wellbeing. All eleven participants believed that dance is a form of exercise (Fig. 1) and can contribute to an exercise regime that is fun to engage in. They believed dance could provide energetic full body exercise and is good for fitness.

*And um, you know I think you use your core strength a lot…. (P4)*

*… the exercise is pretty good when you dance… (P5)*

They also claimed that dance offered a means towards improving mental wellness.

*... it's an exercise [but] also good for your mind …. (P4)*

*… But not only, um, for physical, but also mental, emotional, I've…with my experience, I've seen, including myself, come out of the shell through movement, and dance and stuff like that… (P11)*

Participant P4 said that dance offered a way to vent out frustrations and anger in everyday life and stated she felt very good after the dance session.

*…. you're stamping on the floor…getting angry with the floor…. (P4)*

Extrinsic motivation came in the form of other peoples' opinions, suggestions, and reviews. Two participants got involved with dance-based exercise because someone (roommate, family members, school-based instructor) introduced them to it.

*Well, I first came across it when I was, um, with my nieces and nephew, and it was basically something that we were doing together as a family that we were doing over the Christmas holidays and we had a lot of fun doing that. (P2)*

*I probably did aerobics a bit in high school. (P2)*

All eleven participants said that music took their mind off the negative experiences of exercising such as feeling tired and feeling pain and thus they would at the least try out any form of exercise associated with music.

*Well, it helps you relax and makes you feel ... It takes your mind off the pain of what you're doing… (P9)*

Ten participants said that music played an important role in their exercise regime and they listened to music while exercising (Fig. 1). Eight participants took to dancing as an exercise because of positive experiences with the music associated with the type of dance. For example, one participant enjoyed aerobics when it was set to music in comparison to an older form of aerobics.

*So, like back in ... when I was in my forties ... when I started going to exercise classes like aerobics in my early thirties, mid-thirties, they were very like star jump, star jump, run around the room which I didn't enjoy but when music or dance orientated aerobics came in, I really enjoyed it. (P4)*

Another participant suggested that the type of music played determined whether the experience of dancing and exercising was good or not.

*… probably the fact that it was Latin, so the music was fantastic… (P2)*

The co-occurrence tool applied to Sustained engagement with Dance and Sustained engagement with exercise resulted in three attributes for sustained engagement with dance as a form of exercise: fun; skill, experience and confidence in dancing; and social setting. Six Participants stayed with dance-based exercise because they just enjoyed it. Four participants remained engaged with dance-based exercise because they had the skill or/and experience with dancing. Participant P4 had learned Flamenco and various other forms of dance and at the time of the study was enrolled in a Tango class. The skill and experience made her feel confident about her skills. Participant P8 had picked up dance at school and, although not currently engaged, was keen to learn more dance styles/take up dance-based exercise because she thought she was good at it.

*At school, many moons ago…I was very good at it. Received an award for a dance performance…P8*

The confidence was combined with the fact that she also thought that dance would provide full body exercise.

*…Um, because I think it's a good form of exercise because you're using your whole body. (P8)*

Four participants suggested that the social aspect was critical for staying on track with their exercise regime. They claimed that the social setting facilitated motivation and fun in their exercise routine which made them stick to their routine on a regular basis.

*Ahhh, it was uh ... it was like being motivated, yes and having the whole ... so many people in the building, all of us doing it together... (P4)*

Four participants suggested that it did not matter what type of dance they were engaging in as long as they were doing it with their friends.

*Silly dancing, I suppose, [laughs] yeah I like, just social dancing (P5)*

The co-occurrence tool applied to dis-engagement with Dance and dis-engagement with exercise resulted in three attributes for dis-engagement with dance as a form of exercise: Lack of experience, skill and confidence in dancing; problems with social settings; logistics of attending dance classes. One participant reported disengaging from dance-based exercise due to injury/health related issues. The perception with dance as a form of exercise is that it requires skill and learning. If either or both are not facilitated, people could disengage from the dance-based exercise due to lack of confidence resulting in lack of motivation. One participant did say that she did not feel confident about her dancing skills.

*I am shockingly bad at it...not graceful at all. (P2)*

Lack of skill in dancing becomes more prominent in social settings, and could make people feel embarrassed to dance in front of others.

*Like I said I am not very coordinated,* *laughs*, so going to classes and stuff like that I don't like doing that, because I get self-conscious and feel stupid. (P2)

Dancing in a social setting could bring other challenges. Different people have different preferences regarding who to dance with, type of music played during the exercise, age groups of people in the social setting and so on. Participant P5 had found a group of women of her age to exercise with.

*… it's mostly women my age, or fifties, couple of younger ones…. (P4)*

However, she found the women very noisy and chatty. She could not hear the music that was being played. She remained engaged for a while because the exercise was giving her a good workout, but left the group after one month.

In a social setting involving dance as a form of exercise, people were specific about who they danced with. Negative experiences, such as the group being noisy as in the case of participant P4, could result in disengagement with the dance-based exercise. Participant 2 preferred to dance with her husband but her husband is not interested in dance. This resulted in her giving up dance-based exercise and talking up walking instead as a form of exercise so that she could do it with her husband. This indicates that in a social setting, people may want to look at a form of exercise that allows them to spend more time with their family/friends.

Two participants disengaged from their dance-based exercise due to logistical issues posed by location and timing of group dance settings and the cost associated with dance-based exercise. Both the participants believed that they needed to go to a class to participate in a dance-based exercise as they needed professional help to do the routines properly. Thus, the logistical issue is tied to the fact that some people believe that dance requires skill and learning and that it is something that cannot be done on their own.

*...Convenience is a big thing for me …. So, if it was there and it was not so expensive I would consider it (P3)*

### 6.2 Engaging with SSTs in the context of dance-based exercise

Participants used SSTs such as apps, controller apps with gaming consoles, DVDs, monitors and trackers, and pedometers with the prime objective of keeping fit and healthy. The interpretation of most participants when asked about SSTs was that we were asking about apps. Most apps available in the market are meant for tracking progress and thus the participants' understanding of SSTs was that they are technology based gadgets that help monitor and track progress during an exercise regime. Thus, the participants were probed with questions to reflect on their potential use of SSTs in exercise. The attributes contributing to engagement, sustained engagement and dis-engagement with SSTs for dance as a form of exercise are summarised in Table 4 and discussed below.

**Table 4:** Attributes contributing to engagement, sustained engagement, and dis-engagement with SSTs for dance as a form of exercise.

Attributes for point of engagement with SSTs for dance-based exercise	Intrinsic Motivation – to track progress
	Extrinsic Motivation – recommended by others
	Convenience of transporting and storing the SST.
	Novelty of new technology.
Attributes for sustained engagement with SSTs for dance-based exercise	Time, money, and effort investment.
	Positive results from the exercise routine.
	Motivation of setting goals, challenges, and competitions.
	Positive experiences from the interface.
	Convenience of use and setup
Attributes for dis-engagement from SSTs for dance-based exercise	Negative results from the exercise routine.
	Lack of convenience of carrying, storing and configuring SST
	Novelty fades
	Annoying features of SSTs

The co-occurrence tool, when applied to the three themes Engagement with Dance, Engagement with exercise, and Engagement with SSTs revealed four attributes for engagement with SSTs for dance as a form of exercise: intrinsic motivation; extrinsic motivation; convenience of transport and storage; and novelty of new technologies.

One of the reasons for people to engage with SSTs for dance-based exercise was because someone (e.g. children in the family, roommates) introduced them to it. Another reason was to track and monitor the progress in a graphical and visual format. One participant suggested he started using apps to view his progress in the form of pretty graphs and statistics.

*…then I wanted to have pretty graphs…(P1)*

Convenience was another factor that encouraged participants to use SSTs as they could just carry a device to track progress and view their status.

*…I used to have a notepad to write everything down on…but I like to see how I was doing without having to like flip through all the pages…(P1)*

Novelty of new technology and features is another attribute that engages people with SSTs.

*Just bought a new phone, so it was an initial craze kind of… (P5)*

The co-occurrence tool when applied to the three themes: Sustained engagement with Dance, Sustained engagement with exercise and Sustained engagement with SSTs resulted in five attributes for Sustained engagement with SSTs for dance as a form of exercise: investment of time, money and effort; positive results from the exercise; motivation of setting goals, challenges and competitions; positive experiences from the interface; convenience of use and set-up.

Effort and time required to configure and set up any new SST (such as installing a new app) can result in people preferring to continue using a SST that they have already engaged with. Three participants suggested that they continued using the SSTs (sustained engagement) because it was technically painful to switch between technologies.

*Um, I guess switching from like one app to another is pretty painful, you basically have to start again… (P1)*

People tend to continue using a technology once they have invested time, space, and money in it. Using a new SST could also mean losing tracked and monitored data. However, this sustained engagement could persist only if people can derive positive results from the exercise. Participant P2 associated the successful positive outcome of the exercise regime with the use of SSTs for tracking and monitoring.

*…I was getting fit doing it…. (P2)*

The next attribute is the feasibility of SSTs in allowing users to set goals and motivating users through challenges and competitions to be incorporated in the exercise routine. Participants continued using apps because they allowed them to set goals, and they were rewarded when the set goals were achieved.

*just so it sets some, like, goals for me. And so it sets some benchmarks…(P3)*

*if you get so many steps there's a reward at the end…(P9)*

This attribute is linked to the attribute of positive outcomes from the exercise routine. If the SST can challenge and motivate the user to keep exercising, this should in turn result in positive outcomes, rewards, and thus sustained use of the SST. On the other hand, if the goals are not met and the user is not rewarded, the user may dis-engage.

A well-designed interface generates positive experiences during the use of SSTs, which can help users to remain engaged with the SST. Positive experiences with SSTs as stated by participants were experiences associated with their interactions with the SST interfaces.

*Like you are not kind of having to dig around in crappy interfaces and that kind of stuff… (P1)*

Convenience in terms of where SSTs can be used and effort required to setup the device before use was an important attribute to three participants. One participant preferred easy setup mechanisms such that it is convenient to use at any time.

*…I don't like going anywhere when I exercise. It has to be a spur of the moment, 'I could exercise now', alright let's exercise. It has to be right there. When I have the moment, when I can be bothered, it has to be really flexible and completely under my control…I don't want to rely on anyone else. (P10)*

Participants also expressed their preference towards SSTs which require less manual intervention and are automatic.

*I don't have to do anything, really. It's all done for you… (P7)*

The co-occurrence tool when applied to the three themes: Dis-engagement with Dance, Dis-engagement with exercise and Dis-engagement with SSTs revealed four attributes for dis-engagement from SSTs for dance as a form of exercise. These were; negative results from the exercise regime, lack of convenience in storing, configuring and transporting SSTs, novelty fading, and annoying features of SSTs.

Lack of convenience in terms of space occupied by the SST, space required for the use of the SST and the setup configuration required before use (such as in gaming consoles) could result in disengagement. This was evident in the case of participant P2 who found setting up the Wii for using Just Dance cumbersome and had storage issues with the Wii fit.

*…it was a bit of a pain…having [to] boot the Wii up and drag the steppy thing out and all that kind of stuff…(P2)*

Four participants stopped using SSTs because of negative results reported by the SSTs. Participant P2, for example, stopped using an app because it consistently reported lack of progress in terms of reaching number of steps.

*…It changed the little icon to make you look fat. That got too depressing…(P2)*

People engaging with SSTs due to the novelty of technology and/or brand could stop using them as the novelty fades, which could also be exacerbated by the other attributes of disengagement discussed above.

*…in the short term yes because it was something that was a bit of a fad but it was not something that I…found was maintainable…(P2)*

Another attribute that concerned two participants was an annoying feature, in both cases the narration and voice of the narrator in the SST.

*I don't think I did not enjoy anything in SSTs. Maybe the voice (laughs) actually…It drives my husband insane, the voice. (P11)*

*Yeah and um, but it annoyed me because I didn't like, you know ... be doing the exercise and this voice go. Oh that's good ...... You know that's good. And I just go shut up and it put me off. (P4)*

## 7 Discussion

Generally, our findings confirmed the model of engagement proposed by O'Brien and Toms, with attributes found within the point of engagement, sustained engagement and dis-engagement. This framework was a useful and appropriate approach to interrogating our data. Our results suggest that dance is perceived as an important form of experience for physical, as well as mental and emotional, wellbeing. However, for people to take up dance as a form of exercise, learning and development of dance skills might be required. Developing these skills is important to instill confidence, which in turn is required for sustained engagement with the exercise regime. People generally want to engage in activities that make them feel adequate, capable, effective, and in control (Ryan and Deci, 2000), and previous successes or mastery experiences can also provide positive reinforcement (Gao et al., 2013). Therefore, designing SSTs to facilitate these experiences should increase engagement. For example, because learning of dance steps is important for people who have no prior experience of dance, SSTs should facilitate that learning.

The social aspect is important in many forms of exercise, particularly dance (Domene et al., 2016), and our results support this perspective, with people clearly preferring to dance with a partner or in a formal class setting. People are more likely to adhere to their exercise regime and goals if they exercise in a group (Beauchamp et al., 2007). However, people may prefer to exercise alone when it comes to dance-based exercise if they do not have skills, experience, and/or confidence to dance in front of others (Palo-Bengtsson et al., 1998). Researchers have also suggested that a greater proportion of older people prefer to exercise alone with some instruction rather than within group or class-based settings (Wilcox et al., 1999). Thus, preference to exercise in a social setting could depend on age and further investigation in this direction is required. SSTs could allow users to participate in a social setup, not simply sharing steps or status on social media but participating in a ritual with others or learning to dance with a partner. However, they should also allow the flexibility of dancing alone.

Music was highlighted as a facilitator of dance-based exercise within this study. All participants agreed that music gets them to take their mind off the effort of exercising, with ten of the eleven participants listening to music while exercising. Music has been shown to trigger movement (Murrock and Higgins, 2009) and should be essential for dance-based exercise. Consideration of suitable music styles and options for users to select preferred music are some directions to be explored in the design and development of SSTs for danced-based exercise.

Literature has shown that disengagement may occur if a website is too difficult to use (O'Brien and Toms, 2008), or a text is too complicated or unfamiliar (Douglas and Hargadon, 2000). Similarly, our study showed that poor usability could contribute to disengagement. Previous research has also shown that features unrelated to the core function of a device, including set-up and maintenance routines as well as over-featured interfaces and un-needed functions, can cause negative responses in users (Blackler et al., 2016; Rust et al., 2006), and that ordinary men and women do not use all the complex features that are offered by current self-tracking apps (Didžiokaitė et al., 2017). Results from a study of five popular dance-based exergames showed that games that had a higher usability rating and increased energy expenditure reflected highest on engagement scores and were thus more likely to be used again (Bronner et al., 2015). Our study showed that easy to use interfaces helped to keep people engaged, confirming these previous findings and highlighting the importance of careful feature inclusion and design in order to maximise engagement.

Ironically, knowing that a different system may be difficult to set-up and learn did encourage some people to stick with what they had, which suggests an opportunity for well-designed products to stand out in this marketplace, leveraging their usability as a point of difference and to build loyalty. However, at the end of the day, participants wanted the exercise regime to show results, and that was a major factor in them staying engaged with it and with the associated SST. Ryan and Deci (2000) found that receiving positive feedback enhanced intrinsic motivation, while negative performance feedback resulted in diminished intrinsic motivation, and our findings have borne this out.

Our results suggest that SSTs for dance-based exercise should be simple to use, always available, and require minimal set-up routines. Users should be able to participate in an exercise regime anytime and anywhere as per their convenience. Logistical problems were a major reason for disengagement and SSTs have the potential to overcome these problems almost completely (e.g. users can set their own time and place, involve who they like and pay out less money over time).

SSTs could offer greater scope than purely progress tracking devices. People believe that dancing requires skill and training, which is something SSTs can play an important role in providing. SSTs could, for example, provide training for certain dance steps or help partners to learn how to dance together. Careful design of products and services which facilitate realistic goal setting and maintain engagement with dance-based exercise can assist to make this happen – for example, through structuring of weekly targets related to learning dance steps and/or prompts to practice what has been learnt to-date, subtle reminders about posture and movement during complex dance moves, or through pushing interesting new music and dances to the system periodically. These sorts of features may be far more attractive and useful to ordinary men and women than the kinds of extras offered by many fitness apps (heart-rate, sleep tracking, calorie counts, etc).

Overall, our findings show that SSTs have a role in facilitating movement and increased engagement with physical activities if they facilitate dance based exercise that also supports people's confidence, dance knowledge and positive experiences.

## 8 Conclusion

This study has shown that dance can be a form of enjoyable exercise, and that there is appetite for SSTs for dance-based exercise. SSTs can play an important role in dance-based exercise, however their role needs to go above and beyond simple tracking. The study has revealed that people's engagement with dance as a form of exercise as well as use of related SSTs is governed by positive results or feedback from both the exercise regime and the SSTs. The role of SSTs should thus primarily be facilitating dance and exercise rather than just reporting and tracking. Domene et al. (2016) explain how adherence to a dance exercise regime is likely maintained only when participation is enjoyable, offers opportunities for mastering new skills, facilitates a self-regulated experience, and provides connectedness with others. SSTs need to provide learning and feedback, be able to eliminate logistical problems by adapting to the environment in which the dancing is performed, and facilitate the social side of dance as well as allowing for solo or collaborative remote dance experiences. They can facilitate all of this if we design them appropriately. Our future work in this area includes investigating how different types or groups of people may engage differently with SSTs for exercise, how innovative SSTs can be used to increase physical activity in young children, and the place of dance in facilitating this.

## Declarations

### Author contribution statement

Alethea Blackler: Conceived and designed the experiments; Analyzed and interpreted the data; Contributed reagents, materials, analysis tools or data; Wrote the paper.

Shital Desai: Analyzed and interpreted the data; Contributed reagents, materials, analysis tools or data; Wrote the paper.

Levi Swann: Analyzed and interpreted the data; Wrote the paper.

Marianella Chamorro-Koc: Conceived and designed the experiments; Contributed reagents, materials, analysis tools or data.

Gene Moyle: Conceived and designed the experiments; Wrote the paper.

Mikaela Stephens: Performed the experiments; Analyzed and interpreted the data; Wrote the paper.

### Funding statement

This research did not receive any specific grant from funding agencies in the public, commercial, or not-for-profit sectors.

### Competing interest statement

The authors declare no conflict of interest.

### Additional information

No additional information is available for this paper.
