# Supplementary material for: Using technology to enhance and encourage dance-based exercise
Source: Heliyon. 2019 Mar 7;5(3):e01241. doi: 10.1016/j.heliyon.2019.e01241 (PMC6407087; doi:10.1016/j.heliyon.2019.e01241)
Supplement: Appendix A [file mmc1.docx]

| **Question number** | **Question phrasing** |
| --- | --- |
| Q1 | How regularly do you exercise? |
| Q2 | Do you use any forms of technology or services to assist with your exercise or wellness? |
| Q2a | Have you ever used any in the past? |
| Q3 | In general, what do you know about self-service technology? |
| Q4 | What SSTs do you currently use? |
| Q5 | How often? |
| Q6 | And how long did you use them for? |
| Q7 | What made you choose that service and that technology? |
| Q8 | What did you enjoy about using that service? |
| Q9 | So what did you least enjoy about that service? |
| Q10 | Why did you cease using them? |
| Q11 | What do you think would help you maybe re-engage with that tech and service? |
| Q12 | Did you believe that the tech/service was effective in assisting your exercise? |
| Q13 | Do you have any gaming consoles in your household? |
| Q14 | Do you use any of those consoles regularly now? |
| Q15 | Do you own/use any movement or exercise related games? How regularly? |
| Q16 | In your own opinion, would you consider dancing as a form of exercise? |
| Q17 | Are you involved in any dance related exercise at the moment? |
| Q18 | Have you ever been involved in any dance related exercise? |
| Q19 | Did/do you enjoy it when you participate? |
| Q20 | So what would motivate you to choose dance as a form of exercise? |
| Q21 | Do you believe any technology could motivate you to choose dance as a regular form of exercise, if at all? |
| Q22 | What are/were the types of dance that you enjoy? |
| Q23 | Do you prefer to dance alone, or with a partner? |
| Q24 | Do you listen to music while you exercise? |
| Q25 | Do you feel music enhances your mood, and why? |
| Q26 | Are you more inclined to exercise if there is music playing? |
| Q27 | What are the specific music genres that make you want to move? |
| Q28 | What other types of social or casual bodily movement do you participate in? |
| Q29 | Considering what we've talked about today, in your opinion what is the most important to you? |
| Q30 | And is there anything we have not talked about today that you think we should have? |
